# NRRS: a re-tracing strategy to refine neuron reconstruction

**DOI:** 10.1093/bioadv/vbad054

**Published:** 2023-05-18

**Authors:** Yiwei Li, Shengdian Jiang, Liya Ding, Lijuan Liu

**Affiliations:** Institute for Brain and Intelligence, Southeast University, Nanjing, Jiangsu 210096, China; Institute for Brain and Intelligence, Southeast University, Nanjing, Jiangsu 210096, China; Institute for Brain and Intelligence, Southeast University, Nanjing, Jiangsu 210096, China; Institute for Brain and Intelligence, Southeast University, Nanjing, Jiangsu 210096, China

## Abstract

It is crucial to develop accurate and reliable algorithms for fine reconstruction of neural morphology from whole-brain image datasets. Even though the involvement of human experts in the reconstruction process can help to ensure the quality and accuracy of the reconstructions, automated refinement algorithms are necessary to handle substantial deviations problems of reconstructed branches and bifurcation points from the large-scale and high-dimensional nature of the image data. Our proposed Neuron Reconstruction Refinement Strategy (NRRS) is a novel approach to address the problem of deviation errors in neuron morphology reconstruction. Our method partitions the reconstruction into fixed-size segments and resolves the deviation problems by re-tracing in two steps. We also validate the performance of our method using a synthetic dataset. Our results show that NRRS outperforms existing solutions and can handle most deviation errors. We apply our method to SEU-ALLEN/BICCN dataset containing 1741 complete neuron reconstructions and achieve remarkable improvements in the accuracy of the neuron skeleton representation, the task of radius estimation and axonal bouton detection. Our findings demonstrate the critical role of NRRS in refining neuron morphology reconstruction.

**Availability and implementation:**

The proposed refinement method is implemented as a Vaa3D plugin and the source code are available under the repository of vaa3d_tools/hackathon/Levy/refinement. The original fMOST images of mouse brains can be found at the BICCN’s Brain Image Library (BIL) (https://www.brainimagelibrary.org). The synthetic dataset is hosted on GitHub (https://github.com/Vaa3D/vaa3d_tools/tree/master/hackathon/Levy/refinement).

**Supplementary information:**

Supplementary data are available at *Bioinformatics Advances* online.

## 1 Introduction

Digital tracing or reconstruction (Meijering, 2010) of a neural morphology is defined to geometrically model the backbone of neurites with countable topologically connected structure components like 3D spheres or lines segments (Gillette *et al.*, 2011), and stores the coordinate, thickness and graph connectivity information in SWC file (Stockley *et al.*, 1993). In the context of digital tracing or reconstruction of neural morphology (Basu *et al.*, 2016; Huang *et al.*, 2022; Liu *et al.*, 2022; Peng *et al.*, 2011a,b; Xiao and Peng, 2013), editing refers to the process of refining the results to ensure accuracy (Meijering, 2010; Peng *et al.*, 2011a,b). This can involve manual proofreading and refinement of the raw tracing results to fit the centerline of neurites imaging signals. Additionally, various techniques have been developed to improve the accuracy of reconstruction results, such as using higher resolution imaging techniques (Economo *et al.*, 2016; Gong *et al.*, 2016), developing more sophisticated algorithms for automatic tracing, and incorporating machine learning approaches etc. (Bria *et al.*, 2016; Peng *et al.*, 2017; Wang *et al.*, 2019a,b; Zeng, 2018). The goal of editing is to provide a detailed and accurate representation of the neural morphology, which can be used to better understand the structure and function of the nervous system.

Recent advances in whole-brain imaging and related technologies have led to the development of several complete neuron morphology reconstruction datasets in whole-brain scale. These datasets include Janelia Research Campus’s 1002 neurons’ dataset (Winnubst *et al.*, 2019), SEU-ALLEN’s 1741 neurons’ dataset (Peng *et al.*, 2021), and ION’s 6357 neurons’ dataset (Gao *et al.*, 2022). Fine digital tracing and associated morphological characteristics from these datasets are highly required for various applications, such as classifying neuronal cells, determining the role of single neurons within neuronal circuits (Peng *et al.*, 2013), and electrophysiological simulation of individual neurons (Zhang *et al.*, 2017). However, due to fluctuations in image quality of tera-voxel scale whole-brain datasets, manual editing remains an integral part of the production of gold-standard neuron reconstruction datasets. Human experts are involved in the reconstruction workflow to partially resolve the complex annotation challenges posed by the whole-brain scale. Yet, different degrees of deviation at branches and bifurcation points are produced, which in turn decreases the utility of further extraction of fine structures like dendritic spines and axonal boutons.

**Fig. 1.**
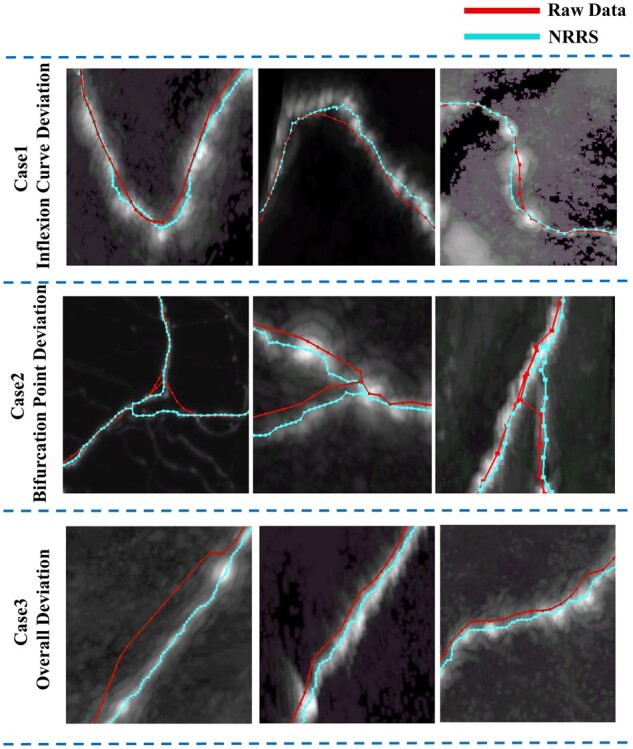
Examples of deviation cases. Case 1: The reconstruction result does not cover the entire bending of the signal. Case 2: The reconstruction result fails to find the accurate position of the branch point. Case 3: Overall deviations of the reconstruction results

The in- or post-process refinement step is crucial in mitigating the deviation of neuron reconstruction and improving efficiency, particularly in regular non-automatic annotation protocols at the whole-brain scale (Peng *et al.*, 2021; Winnubst *et al.*, 2019). To better understand the situation, we have identified three major cases of reconstruction deviations (Fig. 1). Case 1 deviations occur at internal inflexion neurites, particularly in images with a low signal-to-noise ratio (SNR). Case 2 deviations occur at bifurcation points, while Case 3 shows three to eight pixels of directional deviations of entire neurites. Deviations like Case 1 can be caused by a resample operation to a raw SWC file, while Case 2 is mainly due to halfhearted annotation by a human annotator. Both Case 1 and Case 2 can also be attributed to noise from a tracing algorithm with weak tracing performance. A commonly utilized strategy for handling terabyte-sized whole-brain imaging datasets is to create a hierarchy. For example, TeraFly organizes large-scale imaging datasets into multiple resolution layers to provide a navigation experience similar to Google Maps. However, simply mapping tracing results at a low-resolution layer to a high-resolution layer would result in unfitted reconstruction results (Case 3).

Mean-Shift (MS), as an algorithm proposed for shifting nodes to the nearest centroid literately (Comaniciu *et al.*, 1999), is usually applied to refine neurite reconstruction in a local imaging block. One drawback for MS is that a maximum searching distance parameter needs to be provided for constraining the solution space, which is hardly general for a complete neuronal reconstruction crossing thousands of imaging blocks. Other potential solutions to the limitations of Mean-Shift (MS) algorithm for refining neurite reconstruction include 3D shape restriction Mean-Shift (Jiang *et al.*, 2020) and an optimization algorithm based on the Lasso approach (Li *et al.*, 2019). 3D shape restriction Mean-Shift can lead each processing reconstruction unit, typically a node in SWC file, to a locally optimal position without considering the rationality of the entire fitting results. Unlike MS, 3D shape restriction Mean-Shift does not require a maximum searching distance parameter, which makes it more suitable for complete neuronal reconstruction crossing thousands of imaging blocks. The optimization algorithm based on the Lasso approach optimizes the shape of neurons by setting the objective function. This method does not have the problem of selecting MS parameters, but the effectiveness depends heavily on the initial points. In addition, the results cannot change the number of reconstruction points, which limits the final optimization effect to some extent.

To address the deviation cases mentioned above, we introduce a Neuron Reconstruction Refinement Strategy (NRRS). Instead of developing a new optimization algorithm for complete neuronal reconstruction, we focus on automatic tracing methods that have been extensively studied during the past decade. These automatic methods internally contain centerline optimization functions that are perfect for our refinement goal. Based on this idea, our strategy partitions neuron morphology reconstruction into fixed-size segments and resolves the deviation problems through two steps of neuron tracing. Additionally, we build a synthetic image dataset that serves as the validation dataset for quantifying the performance of our method. We show that NRRS has the ability to handle most of the defined deviation errors, while existing solutions hardly achieve matched performance. When applying our method to the SEU-ALLEN dataset containing nearly 2000 complete neuron reconstructions, we present large improvements obtained in the accuracy of the neuron skeleton representation, radius estimation and possible axonal boutons’ detection. The results demonstrate the indispensability of our strategy in refining neuron morphology reconstruction. As a result, we have a version of the results of neuronal reconstruction with more accurate and abundant biological information.

## 2 Materials and methods

### 2.1 Datasets

The study is being validated and tested using ∼1741 complete neuron reconstruction datasets (R1741). These datasets are semi-automatically annotated by the SEU-ALLEN joint center on 34 mouse brains with the fluorescence micro-optical sectioning tomography fMOST. To precisely quantify the feasibility of refinement methods, which have not been done by any related studies, we built a synthetic dataset as a test set to reproduce the possible refinement needs in neuron reconstruction pipelines. The synthetic dataset comprises 1065 synthetic local neural image blocks and their corresponding neuron reconstruction results. The generated workflow contains two parts: the neuron skeleton (synthetic neuron reconstruction results) formation part and the image formation part (Supplementary Fig. S1). After the workflow, the ground truth neuron skeleton data, test neuron skeleton data and synthetic neuron images can be obtained. Furthermore, we randomly sampled 6504 neuron reconstruction 3D blocks (512×512×256, XYZ) from R1741 to further validate the actual performance of our method. Since the whole-brain neural images are too large to be checked in 3D-view manually, we generated the 178 849 MIP (Sato *et al.*, 1998) images (512×512, XY) of the R1741 to check whether our strategy had introduced potential mistakes.

### 2.2 Methods

**Fig. 2.**
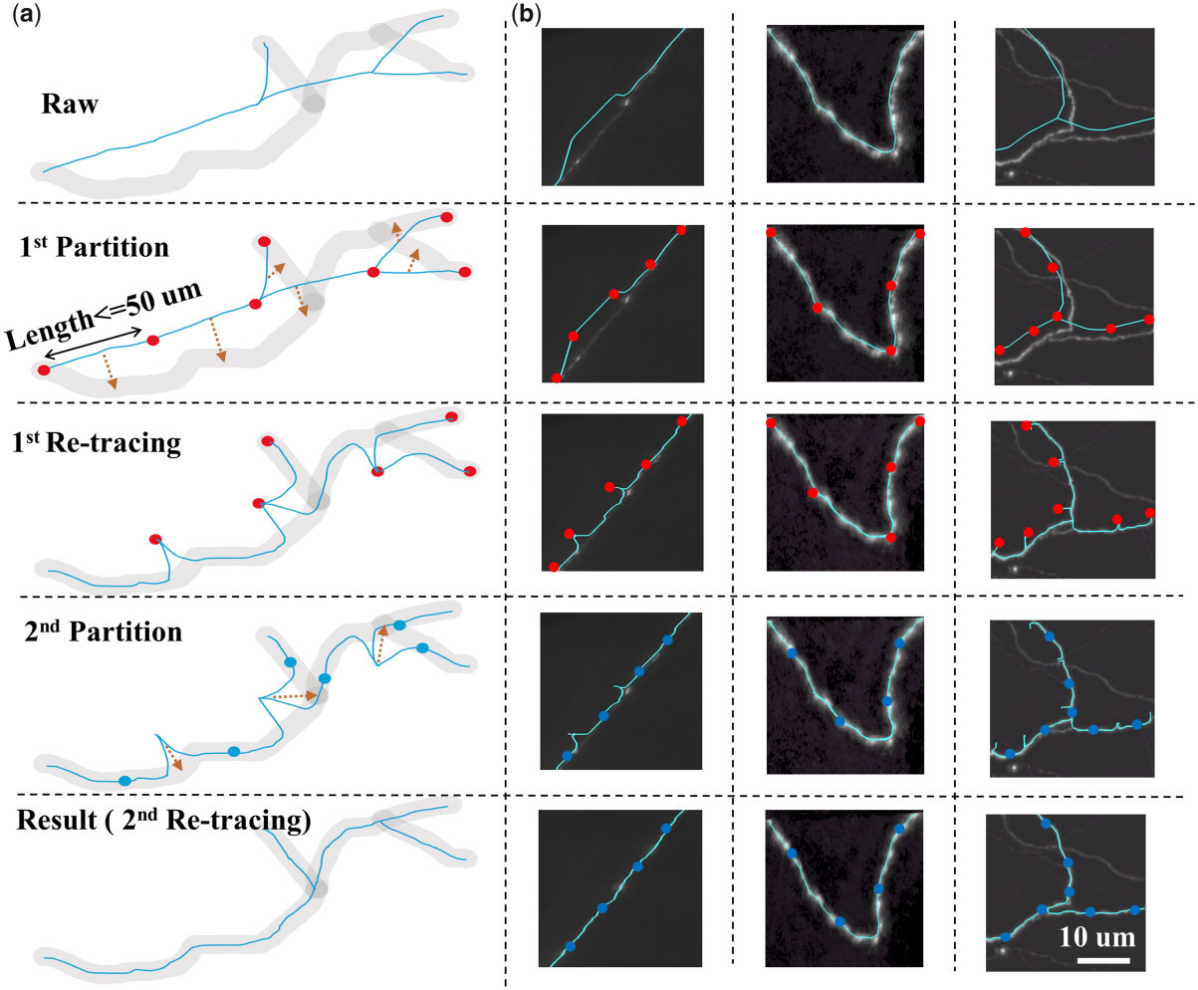
Overall workflow of our refinement strategy. Dots: reconstruction nodes, the connection between which is the neuron skeleton (blue curve) of the segment. Different dot colors indicate results after different partition steps. (**a**) Schematic of the four refinement steps: (i) the first partition step: break each neuron branch into segments less than 50 µm. (ii) The first re-tracing step: trace paths between given points. (iii) The second partition step: find the midpoints of the first re-tracing path. (iv) The second re-tracing step: trace paths between given points. (**b**) The processing results of three neurites in different refinement steps. Scale bar: 10 µm

Our method involves a strategy that consists of two re-tracing steps for dealing with the general deviations of neuron reconstruction, the falsely labeled branching point, and the whole-brain scale challenge. The overall workflow is shown in Figure 2. We assume that the annotation of the starting point (soma) and terminal points is completely correct and limit our refinement to work only on the internal nodes of neuron reconstruction results. This method partitions the neuron reconstruction results into a series of segments with a fixed, computer-handled length to ensure the consistency of image quality beforehand. Then, our goal is to optimize each partitioned segment independently, and we achieve this by making two endpoints an input of a tracing algorithm, which can automatically trace an optimal path between these two endpoints. We can then select another pair of endpoints on the traced optimal path as input to do another tracing in the next step.

Details of the procedures are as follows: In the first partition step, we divide neurons into branches based on bifurcation points. We then break down branches longer than 50 µm into smaller segments. In the first re-tracing step, we use a graph-augmented deformable model (GD) as the tracing algorithm, which consists of two steps: the graph step and the deform-step. The graph step constructs a graph structure based on the segmented neuron morphology, and the deform-step deforms the graph structure to fit the image data.

The graph step: We find the shortest path *P* between the given start and endpoint pairs. For graph *G* = (*V*, *E*), *V* is the set of vertices in the graph and *E* is the set of edges among vertices. Each vertex in *V* represents an image voxel. We define the edge weight between vertexes $v_{0}$ and $v_{1}$, and ensure that the first re-tracing skeleton passes through image voxels with high intensity. The edge weight is calculated as follows:


(1) $$e\left(v_{0},v_{1}\right)=\left|\left|v_{0}-v_{1}\right|\right|*(\frac{\left(g_{I}\left(v_{0}\right)+g_{I}(v_{1})\right)}{2})$$

Where $g_{I}$(.) represents the metric of image voxel intensity. It is defined as:


(2) $$g_{I}=\exp(\lambda_{I}{\left(1-I\left(P\right)/I_{\max}\right)}^{2})$$

Where *I*(.) represent the intensity of the image, then we take advantage of the Dijkstra algorithm (Noto and Sato, 2000) to get the shortest path *P* in graph *G*. $\lambda_{I}$ is the given constant.

The deform-step: we adopt the conventional optimization idea and use the coordinates of the points on the tracing path as input parameters, which are called control point estimations, to optimize the objective function, which is also called the energy function. Firstly, we define the initial control point estimations of the deformable model as {$C_{k}$, *k* = 1,2, …, *K*} from the path *P* in the graph-step. The solution to the optimization problem has become how to find the best control point estimations which have the least value of the energy function. Thus, we apply the local optimization approach to further centralize and refine the skeleton curve *C*. The energy function *E* is defined as follows:


(3) $$E=\alpha E_{\mathrm{image}}+\beta E_{\mathrm{length}}+{\gamma E}_{\mathrm{smoothness}}.$$

Where the *α*, *β* and *γ* are the given coefficients of the energy function (*α* = 1, *β* = *γ* = 0.2 in this step). In the first re-tracing step, the most important factor is the image information since the primary goal of is to find the optimal path on the correct signal. Therefore, *α* should be larger than *β* and *γ*. After experiments, we found that (*α* = 1, *β* = *γ* = 0.2) is a good choice.

The energy functions of $E_{\mathrm{image}}$, $E_{\mathrm{length}}$ and $E_{\mathrm{smoothness}}$ are defined as following:


(4) $$E_{\mathrm{image}}=\sum_{k=1}^{K}\left(E_{I}\left(k\right)+E_{c}(k)\right)$$

(5) $$E_{\mathrm{length}}=\sum_{k=1}^{K-1}{\left||C_{k}-C_{k-1}|\right|}^{2}$$

(6) $$E_{\mathrm{smoothness}}=\sum_{k=2}^{K-1}{\left||C_{k}-\frac{C_{k-1}+C_{k+1}}{2}|\right|}^{2}$$

We set two energy terms$\;E_{l}\left(k\right),E_{c}\left(k\right)\;$utilizing the information from the intensity of image and the distance between the skeleton and the center of the signal:


(7) $$E_{I}\left(k\right)=\lambda_{I}{\left(1-\frac{I\left(C_{k}\right)}{\max I\cdot \left[\Theta \left(C_{k},r\right)\right]}\right)}^{2}$$

(8) $$E_{C}\left(k\right)=\frac{\lambda_{C}\Sigma_{q\in \Theta \left(C_{k},r\right)}{\left|\left|C_{k}-q\right|\right|}^{2}I(q)}{\Sigma_{q\in \Theta \left(C_{k},r\right)}I(q)}$$

Where *k* is the node in the shortest path *P*.

After the first re-tracing step, the main body of the segment is matched with the centerline of the neurite signal while two endpoints still deviate. The accuracy of the first tracing can be guaranteed by the GD algorithm’s superiority (Peng *et al.*, 2010). Therefore, in the second partition step, we partition the results of first re-tracing step at the middle points and use the adjacent middle point pairs as the input of the second re-tracing step.

After the first retracing step, the main body of the segment is matched with the centerline of the neurite signal, while the two endpoints still deviate from the neural signal. The accuracy of the first tracing can be guaranteed by the GD algorithm’s superiority (Peng *et al.*, 2010). Therefore, in the second partition step, we partition the results of the first retracing step at the middle points and use the adjacent middle point pairs as the input of the second retracing step.

Next, we change the coefficients of the energy functions in the second re-tracing step. Since the start and end points of the re-tracing algorithm are guaranteed to be on the correct signal, the smoothness and overall length of the neural skeleton become more important, meaning that *β* and *γ* should be larger. Through experimentation, we set *α* = *β* = *γ* =** **0.5. Based on this, a different path is traced through the given points of the second partition and optimized by minimizing the energy function. After the second re-tracing step, the entire skeleton of the reconstructed neuron is refined to the centerline of the neural signal.

## 3 Experiments and results

### 3.1 Validation on synthetic dataset

**Fig. 3.**
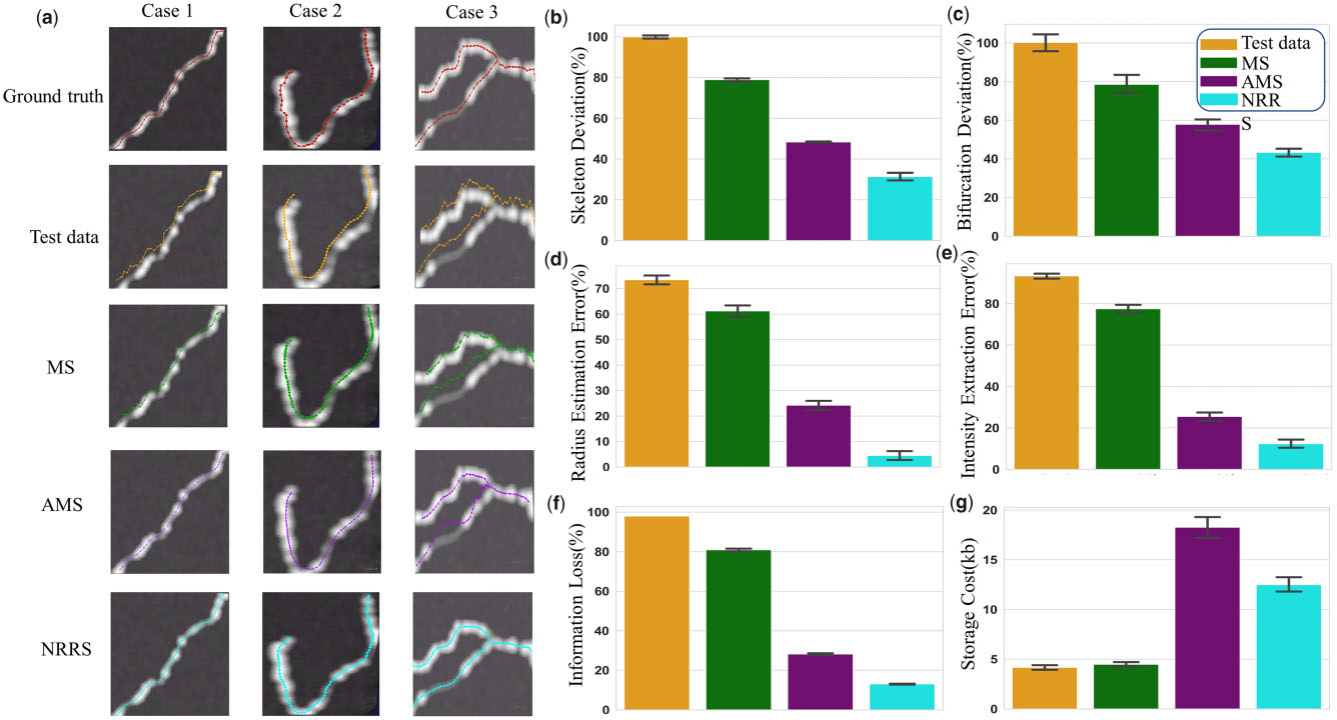
Quantitative validation on a synthetic dataset. (**a**) Examples of the synthetic dataset and the performance of different algorithms. (**b–g**) The quantitative comparison between our strategy and other solutions. NRRS significantly improves the accuracy of neuron morphology reconstruction. NRRS outperforms other solutions in terms of statistics validation for solving the deviation of skeleton and bifurcation points. Additionally, NRRS can obtain more accurate radius estimation and intensity extraction, with less information loss. Moreover, the reconstruction results refined by NRRS have a relatively smaller storage size

To quantitatively assess the performance of existing neuron refinement methods, we compared our method mainly to MS and AMS (a kind of Mean-shift that can automatically choose its parameters, more information can be seen in the Supplementary Materials) on the synthetic image dataset (Fig. 3). The results show that our method outperforms MS, especially at the bifurcation points (Case 3).

In more detail, the first row in Figure 3a shows three types of image blocks from the synthetic dataset that serve as ground truth data. The second row consists of test data consisting of three representative error cases. As shown in the MS row, MS fails to refine the test data to its image signal centerline and also has poor refinement performance at the bifurcation points (Case 3). In contrast, our method works well in all three cases, especially in Case 3, where our method reaches a more reasonable global optimal path than other methods.

We used radius and intensity as important evaluation metrics to assess the performance of our method (Fig. 3). The calculation of these metrics can be seen in the Supplementary Materials. The quantities were calculated on individual synthetic image blocks and collected from 6504 blocks for bar plots. The metrics we used include skeleton deviation, bifurcation deviation, radius deviation, intensity deviation, information loss rate and storage size. The increase of the radius and intensity can serve as important indicators of performance improvement, as the closer the reconstruction results are to the center of the signal, the larger the estimated radius will be and the brightness of the image block where the reconstruction point is generally also larger. All the standards are based on the comparison with ground truth. Our proposed NRRS significantly improves the accuracy of neuron morphology reconstruction (Fig. 3b–g). Our method outperforms other methods in terms of skeleton deviation, bifurcation deviation error and the extraction of radius and intensity information from neurite signals. Specifically, our method decreases the average skeleton deviation to 27% (Fig. 3b) and reduces the bifurcation deviation error to 42%, while other methods only decrease the bifurcation deviation error to 58% (Fig. 3c). Additionally, our method has the most accurate performance in the extraction of radius and intensity information from neurite signals (Fig. 3d–g). Although using AMS can also improve the quality of reconstruction, it requires a much larger storage size and is not always effective or valid. AMS achieves the current performance by sacrificing the cost of storage by adding more reconstruction nodes, which can solve some problems in simple cases. However, our NRRS method is more efficient and effective in handling most deviation errors.

The proposed information extraction scheme is crucial in evaluating the digital representation ability of different refinement solutions. The information extraction rate, which is defined as the proportion of the volume covered by the reconstruction result to the actual volume covered by the ground truth signal, is used to quantify the performance of different methods. By resampling the results of different methods to the same interval (2 µm), we calculated the information extraction rate using the method described in Supplementary Materials. Our strategy only lost about 10% of the information, while other methods lost at least 30% of the information on average. Moreover, when each method achieved the best information extraction rate, we compared the storage results of each method and found that the optimization results of our scheme only require slightly more storage than the original data and less than the MS or AMS algorithm.

#### 3.1.1 Improvements in complete neuron reconstruction dataset

To demonstrate the wide applicability of NRRS, we applied it to the manually annotated version of the R1741 dataset. NRRS resolves the deviation problems in two aspects.

First, manual checks remain the most convincing method to justify the performance of different methods. We manually checked the majority of neurons in the R1741 dataset, and our approach addresses the problem of reconstructions that deviate from the signal. In most cases, refined neuron skeletons are closer to the signal centerline, have brighter signals and more bifurcation point positions. To further verify that our method has not introduced new mistakes, we generated 178 849 MIP images. MIP images can be checked much more rapidly, so we had enough human resources to check all the neurons’ images (Supplementary Fig. S4).

**Fig. 4.**
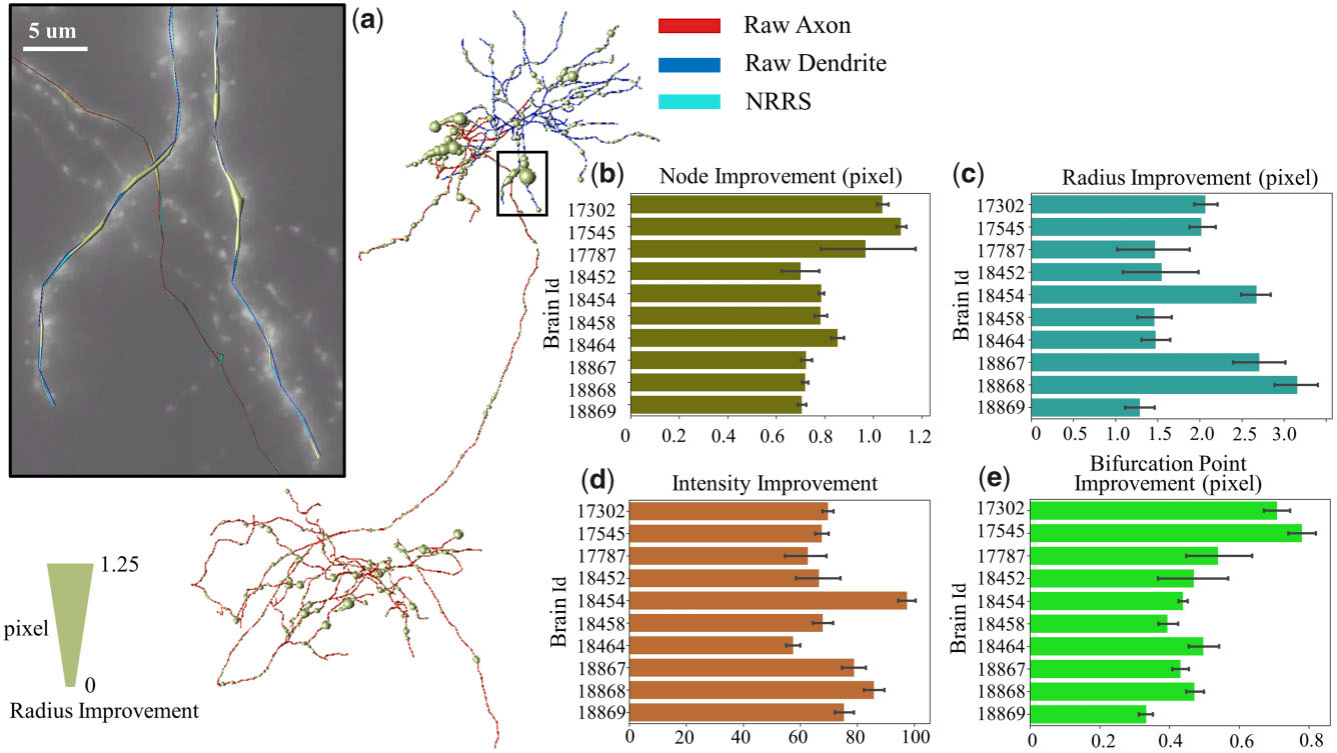
Improvements brought by our method on the R1741 dataset. (**a**) The example shows the improvement in radius extraction on a whole-brain neuron and the zoom-in view on the place with the biggest radius extraction improvement. (**b–e**) The refinement effect brought by our method can be seen in four aspects: node improvement, radius improvement, intensity improvement and bifurcation point improvement

Secondly, through manual checks, we can confirm that our method truly brings improvement, which requires further quantification. For example, we observed larger and more accurate radius estimations (as in the previous part) resulting from our method. Thus, we can define radius improvement as the difference between raw data and refined data. Similarly, in Figure 4, we used four aspects to describe the improvements: node improvement is the average node distance change, radius improvement is the average node radius increase, intensity improvement is the average node intensity increase and bifurcation point improvement is the average distance change of the bifurcation points.

In Figure 4a, the radius of the tube represents the change in node position between the raw neuron and the refined neuron. The dendrites and terminal axons show the most displacement, while the axon main path has an overall shift. In Figure 4b–e, all brains show an enhancement in four features. The average radius has increased by 0.5–1.25 pixels for each brain, indicating that some brains may have had better-reconstructed results previously. The changes brought by the refinement algorithm seem to be more balanced for the average distance change. The average nodes’ refinement distance varies from 1.75 to 2.62 pixels. Our refinement strategy has boosted the average brightness of reconstructed points per brain from 58 to 96 according to the intensity change, indicating that our method has shifted the neuron reconstructed results to the right center of the signal. Lastly, we calculated the average change in bifurcation points, which varied from 0.3 to 0.55 pixels. There are many branch points in a whole-brain-level neuron, and not every bifurcation point needs to be refined heavily. Therefore, the bifurcation offset may not be obvious enough on the average level. A specific example better illustrates this point.

**Fig. 5.**
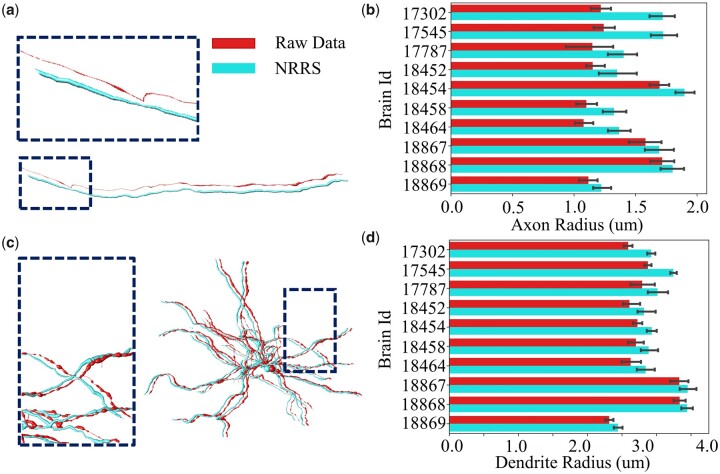
Improvements of radius estimation on axon and dendrite. (**a**) The improvement of the axon radius measurement after refinement. (**b**) Statistical results demonstrate that our method achieves better radius results on over 1400 neurons in 10 whole-brain images. (**c**) Dendrite radius measurement result also has a better performance. (**d**) Statistical results prove that our refinement method has brought an obvious promotion in dendrite radius

We estimated the axon and dendrite radii of the R1741 dataset (Fig. 5). The statistical results show that the average axon radius of nearly every brain has increased by 0.15 µm. For brains 17 302 and 17 545, the peak increase of the average axon radius is 0.5 µm. With respect to neural dendrites, deviations usually occur on the terminal branches. After refining our method, the dendrite radius of each brain is on average increased by 0.1–0.3 µm. Based on manual judgment and quantitative statistics, our method truly provides a better effect on radius estimation.

#### 3.1.2 Improvements in axonal bouton detection

To demonstrate the effectiveness of NRRS in refining neuron morphology reconstruction, we applied our protocols to the task of detecting axonal boutons. Axonal boutons are typically located along the axonal signal are visually distinguishable from axons in light microscopy data due to their larger radius and greater brightness. Researchers use the radius and intensity information of the axon to determine the position of potential boutons. Our method significantly improves the accuracy of radius estimation and intensity extraction, which are crucial in detecting axonal boutons.

**Fig. 6.**
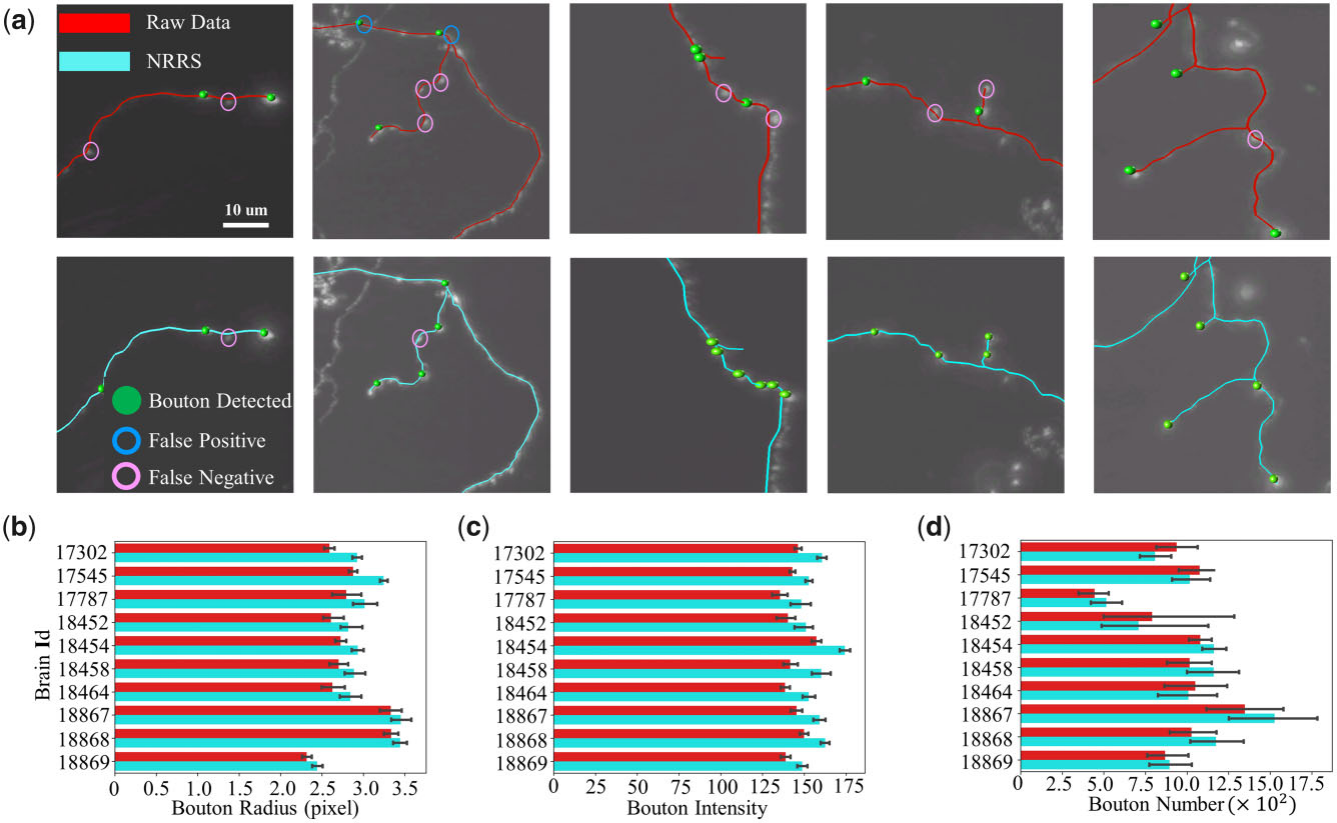
Improvements in whole-brain axonal bouton generation. Red line: raw neuron reconstruction results. Blue line: neuron reconstruction results refined by our method. (**a**) Our method increases the precision of bouton extraction. (**b–d**) Our method extracts the bouton features more accurately and obtains better results on the bouton detection task. The false positive rate increases and the false negative rate decreases

We used the bouton detection function in Vaa3d (Jiang *et al.*, 2022) on the SEU-ALLEN dataset, cropping 305 images to check the results of detected boutons from the neuron skeleton before and after refinement (Fig. 6a, Supplementary Fig. S5). Raw neuron skeletons have many false positives and false negatives. After refinement, however, the more accurate estimation of axon radius led to more rigorous bouton detection results.

We also quantified the boutons extracted from 1741 neurons, including bouton radius, bouton intensity and bouton number. Both the average bouton radius and bouton intensity improved. Statistical results for bouton number show that the number of boutons extracted for some brains decreased. This is because our method not only extracted the originally missed boutons, but also greatly reduced the number of detected erroneous boutons. In conclusion, our method significantly improves the accuracy of bouton detection.

## 4 Conclusion and discussion

Our proposed NRRS significantly improves the accuracy of neuron morphology reconstruction. Our method outperforms existing refinement solutions in either inflexion deviation scenes or entire deviation scenes. In particular, our work has achieved a pretty good performance on refining bifurcation points, which have not been considered by any other refinement solutions. The main goal of our work is to implement a general method not only for partial neuron reconstruction but also for dealing with the ‘big data’ challenge brought by the whole brain scale. We adopted several specific treatments to accomplish this. The first one is a partition of neuron reconstruction so that it can be processed by current computing hardware, and refinement of each partitioned segment would not be affected by changes in image quality. The second one is the re-tracing idea for optimizing varying degrees of reconstruction deviation.

In the validation part, our work formulates a canonical form for the quantitative assessment of the performance of refinement methods and generates a synthetic dataset for validation. Previous studies used visual inspection as the main tactic to validate the refinement performance, which is unrealistic for the ever-growing interest in complete neuron reconstruction at the whole-brain scale. Moreover, validating with the same workflow will greatly improve the speed of refinement works in the development phase and benchmark test in comparison.

We demonstrate the effectiveness of our refinement strategy on the SEU-ALLEN whole-brain neuron reconstruction dataset, which is currently the largest complete neuron morphology reconstruction dataset. Figure 1 shows examples cropped from the SEU-ALLEN dataset, and it is evident that our method significantly improves the accuracy of neuron morphology reconstruction. Our method moves the deviated neuronal skeleton to the center of the signal, even in cases of poor signal quality. In Case 2, our method successfully fits the thin and tortuous signal, and in Case 3, our method accurately identifies the most suitable bifurcation point on the signal, resulting in a more accurate and reasonable neuron morphology. Our strategy reduces the deviation error to less than 6.8% without sacrificing storage size. By using a refined version of this dataset, improvements can be observed in radius estimation, image intensity extraction and axonal bouton detection. Our refinement strategy is a powerful tool for improving the accuracy of neuron morphology reconstruction.

The limitations of present works include two parts: the applicability of the tracing algorithm used in the re-tracing step and the correctness of reconstructed terminal points. Specifically, the GD algorithm used in this manuscript is designed to obtain a path with less defined cost, and different parameter groups for the cost function may output various optimized paths. One set of parameters may not be able to reach a reasonable path for all the situations that occur in whole brain scale. Therefore, one thing that needs to be further investigated is the adaptive setting of parameters. Another limitation is that the current version cannot refine the tract close to soma and neural terminal, since our hypothesis is absolutely correct of soma and neural terminal points. A pre-refine of reconstructed soma and neural terminal could further promote the entire refinement of neuron reconstruction.

Our method is implemented as a C++ plugin in Vaa3d system. The refinement time cost for a neural reconstruction that contains over 30 thousand SWC nodes (the overall reconstructed neurites are about 30 000 µm) is about 3 min on a Linux workstation with Intel(R) Xeon(R) Gold 6132 CPU. And the processing can be tremendously speeded up by parallelization of the re-tracing step, which is highly useful in the large production of neuron reconstruction.

## Supplementary Material

vbad054_Supplementary_Data
