# Supplementary material for: NRRS: a re-tracing strategy to refine neuron reconstruction
Source: Bioinform Adv. 2023 May 18;3(1):vbad054. doi: 10.1093/bioadv/vbad054 (PMC10199312; doi:10.1093/bioadv/vbad054)
Supplement: vbad054_Supplementary_Data [file vbad054_supplementary_data.zip › vbad054_Supplementary_Data/NRRS_supplementary0612.pdf]

Bioinformatics Advances Here, 2023, pp. 1–9

doi: DOI HERE
Advance Access Publication Date: Day Month Year
Paper

PAPER

NRRS: A re-tracing strategy to refine neuron reconstruction

Yiwei Li[1, 2, #], Shengdian Jiang[1, 2, #], Liya Ding[1], and Lijuan Liu1[*],
1 Institute for Brain and Intelligence, Southeast University, Nanjing, Jiangsu, 210096, China
*Corresponding author: lijuan-liu@seu.edu.cn
#Equal contribution
FOR PUBLISHER ONLY Received on Date Month Year; revised on Date Month Year; accepted on Date Month Year

Supplementary Materials

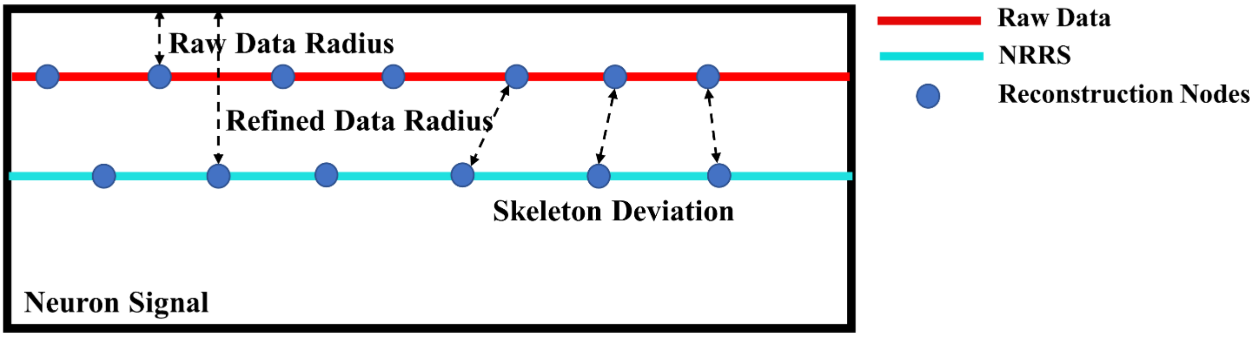

Figure S1. Estimation of Radius and Calculation of Skeleton Deviation. The accuracy of radius estimation depends highly on the position of the neuron skeleton. The closer the neuron skeleton is to the signal centerline, the larger and more accurate the estimated radius will be. The skeleton deviation is calculated by averaging the distance between proper node pairs.

Auto-Mean-shift

Auto-Mean-shift (AMS) is an adaptive Mean-shift algorithm. In many cases, the reason for the failure of Mean-shift is due to improper selection of radius and image threshold, which leads to the inability of Mean-shift to optimize the results to the desired signal center. In this paper, AMS is designed as following. A radius of 5 is selected as the initial radius, and the image is searched according to brightness in the scale of the given radius. If there are fewer image points within the search range than a certain range that are greater than the given threshold, the search radius is expanded until the number of points that meet the brightness requirements within the search range reaches a certain number. Then, the search radius at this time is selected as the radius for Mean-shift, and the mean+std of the brightness of the image block within this radius range is used as the brightness threshold. Points with brightness below the threshold are set to zero, and then Mean-shift is run. It is worth mentioning that the AMS in this paper will perform mask operations on image blocks based on the trend and range of reconstructed points before and after to ensure that Mean-shift will not shift the reconstructed results to other unrelated signals.

Evaluation Metrics

The calculation of the metrics used in the main manuscript to demonstrate the performance of the NRRS is as follows:

The **radiusEstimation** plugin in Vaa3d is used to estimate the radius of signals. This is done by first up-sampling the image to improve accuracy, and then estimating the radius based on the image's brightness at the reconstruction point. The estimated radius will be larger and more accurate if the reconstruction is closer to the center of the signal.

To obtain **skeleton deviation**, the average distance between corresponding nodes of each neuron skeleton from refined data and ground-truth data is calculated. The corresponding node is selected as the node with the least Euclidean distance. Similarly, bifurcation point deviation, radius deviation, and intensity deviation are obtained by computing the corresponding bifurcation point distance, the counterpart nodes' radius, and intensity change between ground-truth data and refined data for each neuron skeleton.

Storage size refers to the average file size of neuron skeletons in the synthetic image dataset. Typically, the more SWC nodes contained in an SWC file, the larger the file size.

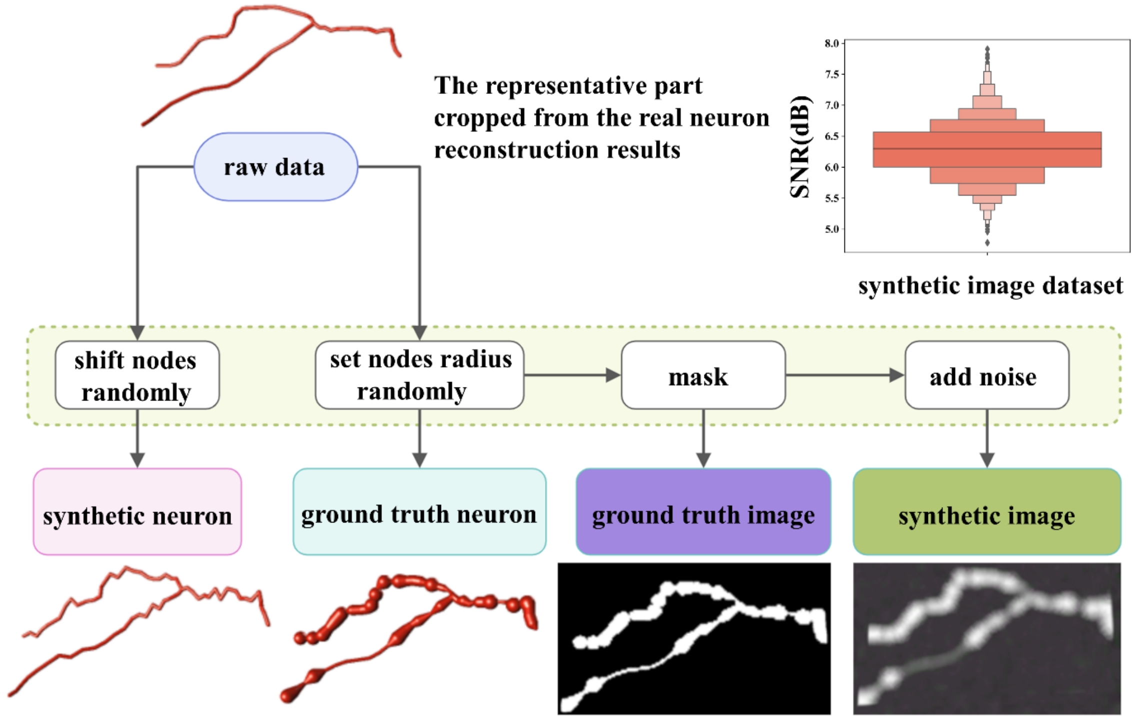

Figure S2. Synthesis of neurons and image production. The synthetic dataset consists of three parts: ground-truth data, synthetic data, and synthetic images.

Synthetic Image Dataset:

To produce synthetic neurons and images, four steps are required.

Step 1: Raw data is cropped from real neuron reconstruction results, and the nodes are shifted randomly to get the synthetic neurons. These neurons are deviated randomly by 1-10 pixels. Since we will do radius estimation on the synthetic neurons, we do not need to define the synthetic neurons' node radii in this step.

Step 2: Each raw data node is given a random radius, which produces the ground truth neurons. The ground truth neuron is used to obtain the overall variance, branch point offset, and radius deviation in the experiments.

Step 3: A ground truth image is obtained by masking the ground truth neurons, and the images are binarized. These images are used as ground truth to calculate the feature extraction rate.

Step 4: Noise is randomly added to the ground truth, and a Gaussian filter is applied to the entire image to obtain the synthetic images. A dataset is composed of these synthetic images to test different algorithms. By calculating the deviation between the ground truth data and the refined neural skeleton results generated by various algorithms, we can gather quantitative analysis on the performance of different methods in this synthetic image dataset.

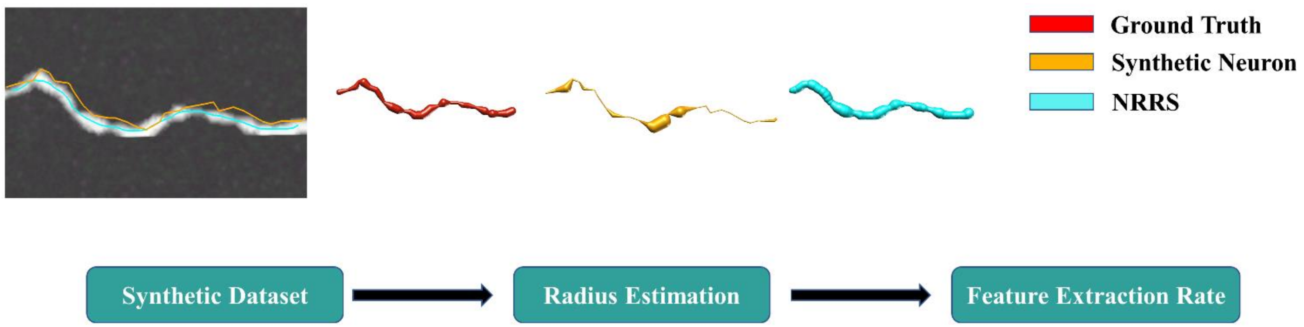

Figure S3. The schematic to calculate feature extraction rate on the synthetic neuron.

The **feature extraction rate**, **r**, is defined as the volume covered by the reconstruction result divided by the ground truth area.

$$r = \frac{V_{refined\ neuron} \cap V_{ground\ truth}}{V_{ground\ truth}}$$

To calculate the information extraction rate, the results of different methods must first be resampled to ensure that the intervals between each node of each neuron skeleton are the same (2 μm). The ground truth area is calculated during the production of the synthetic image dataset, and since the ground truth data's reconstruction nodes have accurate radius information, we can easily obtain

the space that is considered as the neural signal. Similarly, we can use the nodes' positions of different reconstruction results and their estimated radius to obtain the area they cover. The feature extraction rate can then be calculated.

Table 1. The statistical results of all the effects on the SEU-ALLEN dataset.

Brain id	Overall distance (pixel)		Branch offset (pixel)		Radius change (pixel)		Intensity change (0~255)	
	Mean	Std	Mean	Std	Mean	Std	Mean	Std
17302	2.61	0.03	0.68	0.04	0.81	0.06	67.34	2.41
17545	2.67	0.02	0.72	0.03	0.76	0.05	62.18	3.07
17781	3.03	0.05	0.51	0.07	0.32	0.12	48.67	3.97
17787	3.61	0.12	0.53	0.12	0.62	0.13	63.92	5.02
17788	2.37	0.18	0.61	0.03	0.13	0.21	43.52	9.98
18047	2.01	0.21	0.46	0.07	0.17	0.23	45.14	12.23
18272	2.53	0.08	0.57	0.06	0.27	0.07	51.06	3.12
18452	1.78	0.07	0.49	0.08	0.62	0.09	67.38	5.14
18453	1.89	0.02	0.59	0.07	0.25	0.02	56.47	1.96
18454	1.95	0.01	0.43	0.02	1.05	0.03	95.89	2.03
18455	2.04	0.01	0.62	0.04	0.24	0.11	91.97	4.34
18457	1.81	0.01	0.67	0.01	0.68	0.05	75.13	1.37
18458	1.97	0.01	0.39	0.01	0.61	0.06	64.47	3.76
18461	2.63	0.01	0.43	0.03	0.01	0.04	44.31	2.37
18462	2.57	0.01	0.53	0.02	0.09	0.03	59.94	3.12
18463	2.39	0.01	0.57	0.04	0.18	0.04	59.87	2.53
18464	2.15	0.01	0.51	0.05	0.58	0.06	56.73	1.89
18465	1.94	0.01	0.61	0.04	0.49	0.04	70.12	1.52
18470	2.73	0.02	0.31	0.01	0.81	0.12	66.37	6.73
18864	1.74	0.01	0.57	0.04	0.14	0.03	55.13	1.21
18867	1.73	0.01	0.42	0.02	1.12	0.07	77.74	2.57
18868	1.72	0.01	0.45	0.02	1.25	0.06	86.41	3.41
18869	1.71	0.01	0.37	0.01	0.51	0.04	76.12	3.13

MIP

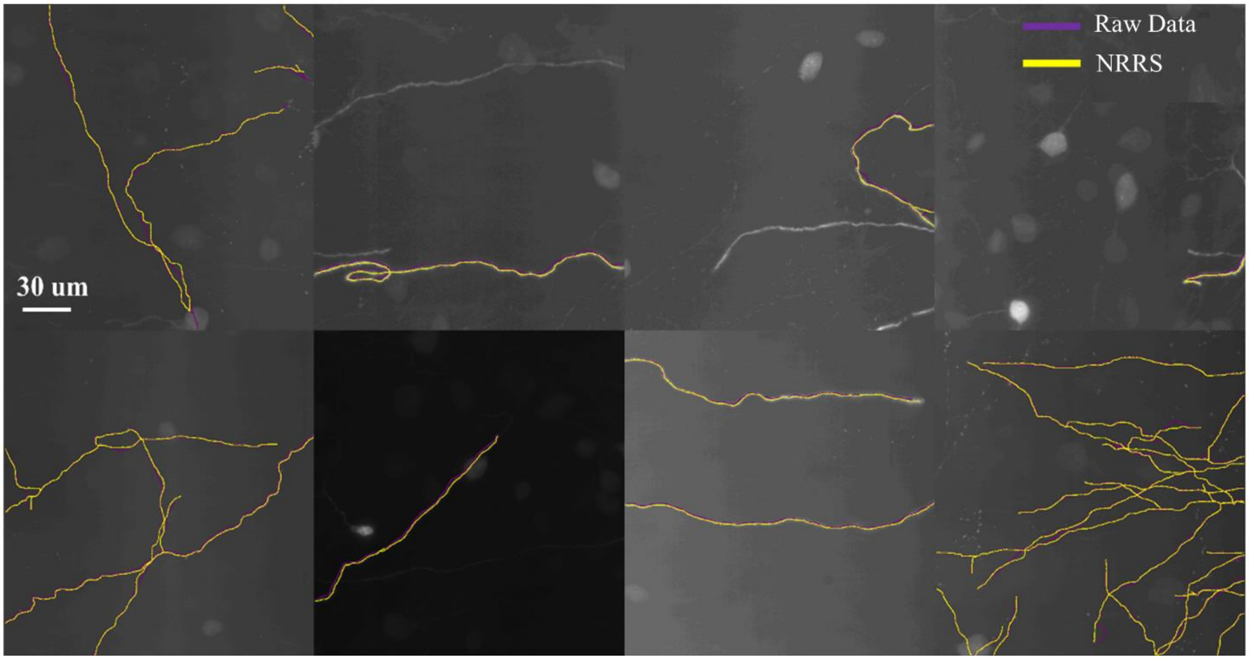

Figure S4. The MIP examples of the SEU-ALLEN dataset. MIP strategy is applied to check the performance of our method on the R1741 full-neuron reconstruction dataset.

MIP Check: To ensure that NRRS does not introduce new errors, we use a MIP strategy to evaluate the performance of NRRS in the SEU-ALLEN dataset. We examined over 178,849 TIFF images **(512x512, XY).**

While NRRS is generally effective, it may make errors when the neural signal is extremely sharp or tortuous. In these cases, NRRS might make shortcut errors that prevent the neural skeleton from covering all the bending curves. During the manual check, we estimated the likelihood of this problem at 2.4%. However, this type of problem does not cause topological errors and can be resolved by adjusting the parameters of NRRS.

Bouton Detection

Examples of **bouton detection**: Here we present additional examples cropped from the SEU-ALLEN dataset. It should be noted that the boutons detected in this article represent only possible bouton positions. According to the experience of neurobiologists, these axonal swellings have a high probability of being axonal boutons. However, due to the limitations of the optical image resolution, this cannot be conclusively determined and is therefore declared as such.

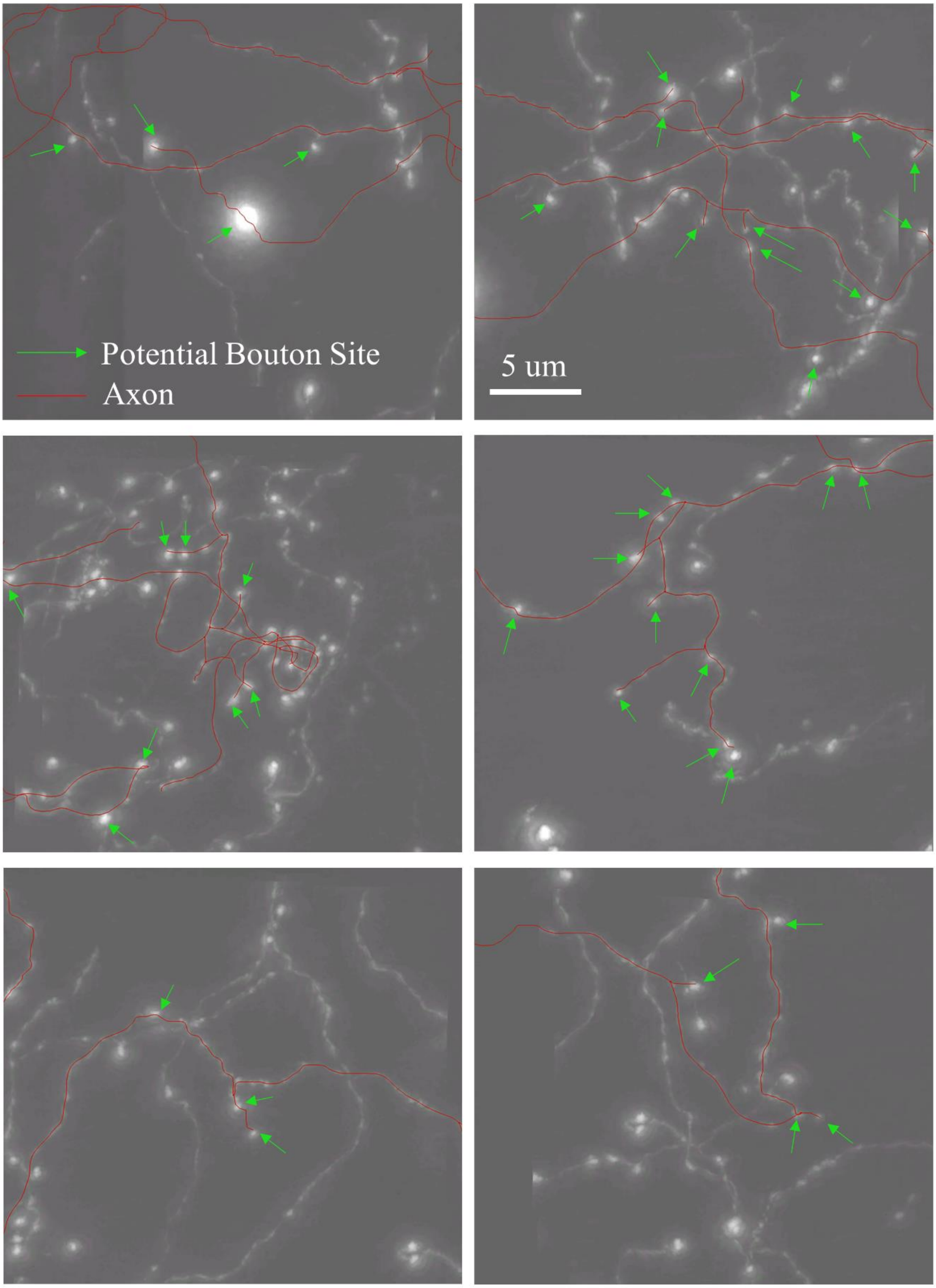

Figure S5. Bouton examples from the SEU-ALLEN dataset. Boutons are axonal swellings that appear as bright, inflated globules in the images.
